# Sulphate of Atropia in Ephidrosis Profusa

**Published:** 1874-05

**Authors:** C. H. Tidd

**Affiliations:** Middleport, Ohio


### SULPHATE OF ATROPIA IN EPHIDROSIS PROFUSA.

BY C. H. TIDD, M.D., MIDDLEPORT, OHIO.

The action of atropia as a narcotic, is too well understood by the profession in general to demand a reiteration at this time, as are also its general therapeutical actions, but there is one property which it possesses, which is not so generally understood, and it is to this that I would call attention in this article, hoping that in so doing I may be enabled to suggest a remedy to my fellow practitioners, that will prove valuable in instances where the usually employed agents have signally failed. I refer to its powers as an "*hydrotic*" and hope to present the subject in a manner at once so concise and clear, that it will recommend itsef to the reader.

The action of atropia in checking the secretion of milk in the parturient woman, naturally led the profession to test its powers, in checking the secretions of other glands as well as of mucous membranes. Dr. Wilhelm Ebstein speaks highly of it as an agent in the treatment of salivation and says: "When I give my patient a hypodermic injection of 0.0016 atropia, I assure him a perfect nights rest, which would be else impossible on his constantly saturated pillow." Sidney Ringer, M.D., in an article in the *Practitioner, Aug.*, 1872, reports his experience in a large number of cases, in which he used the belladonna liniment, externally, with marked benefit. In the course of his investigations he concluded that the agent acted by absoption, and began to administer belladonna internally, but did not derive the same degree of benefit from it when given in this manner, as when applied locally, probably from the fact that when given internally so much less quantity was taken into the system, that the full therapeutical effects of the agents were not obtained. But later in the same year Dr. Ringer began to experiment with the alkaloid atropia, and found equally as good if not much better effects follow its administration (when given hypodermically) than was observed by the local administration of the tinct. belladonna. He says: "Our observations lead us to conclude that $\frac{1}{200}$ of a grain of atropia injected hypodermically, is sufficient in most cases to check sweating for one night."

The writer has in one or two instances checked the sweating of the head of young children, by having them washed in a weak solution of fl. ext. belladonna, say 3 to 5 drops to the ounce of water, and have seen no ill effects follow its use, and the good effects seem so far to be permanent.

Dr. Ringer, in the *Practitioner* says: By rubbing the ointment or liniment of belladonna *in*, two or three times a day, he has checked the profuse sweating of the hands, which prove so disagreeable to the patient.

Mr. C. A. Nawkivell reports the following experiment performed in University College Hospital: "To a middle-aged woman suffering from acute rheumatism, a hot air bath was administered, followed by cold sponging. This caused her to sweat so freely that, for several hours after the perspiration continued to pour down her face, soaking her clothes, and the bed-linen. While in this state, $\frac{1}{100}$ of a grain of atropia was subcutaneously injected into her arm, and in about a minute the perspiration ceased, and for two hours her skin continued dry, and she felt much cooler, but in the evening rather free perspiration returned." He says: We next gave a young man a Turkish bath, and Mr. Johnson, the resident assistant of wards, joined him in the hot chamber, both sweated freely, and then each was injected with $\frac{1}{100}$ of a grain of atropia, and in little more than a minute the skin became dry, and the perspiration did not return upon the application of the cold douche, nor afterwards. Mr. J. remarked, that so dry did his skin seem that he felt he should never sweat again. "They both suffered from dryness of the mouth but their pupils were not dilated." They next placed a boy in the hot air bath, the temperature rising to 180° fahr., and when sweating freely, they injected the $\frac{1}{120}$ of a grain of atropia, and almost immediately the sweating ceased and did not return again."

"They next placed a lad in the hot air bath of 194° Fahr., and when perspiring profusely, injected $\frac{1}{300}$ grain of atropia. In five minutes the perspiration was decidedly less, and in ten minutes was very slight, but in thirteen minutes it again became profuse, they then injected another $\frac{1}{300}$ of a grain, and in two minutes his face became perfectly dry and remained so during the remainder of the bath, *i. e.* ten minutes.

We find also reports from reliable sources, where it has been successfully tried in the night sweats of phthisis and in every case the sleep was better and the cough less troublesome, although generally a disagreeable dryness of the throat followed, but which soon passed off. I deem it unnecessary here to tabulate the cases, as probably most or all of my readers have noticed the reports. Yet there is one farther report which I must introduce at the expense of making this article longer than I had intended.

Dr. Fräutzel,* in a paper on the subject, published in Virchow's *Archir*, vol. 78, part I. Allgemeine Medicin, Central Zeitung, August 2, states "that, having given it to seventy-five patients, he has arrived at the conclusion that it is a remedy which he can confidently recommend, not only in phthisical sweating, but also in that which attends other diseased conditions, such as acute articular rheumatism and convalescence from trichinosis. Among the seventy-five cases were fifteen of more or less recent cheesy pneumonia, of whom all had more or less fever with night sweats; forty-eight of distinct pulmonary phthisis, of whom forty-two had hectic; one of acute articular rheumatism with high fever; two of ulcerative endocorditis, and two of trichinosis. In the first fifteen patients sweating was in six completely arrested; in seven much diminished; in two there was no change. In the forty-eight phthisical cases, the medicine had no effect in five; in twenty-one the sweating was remarkably abated, and in twenty-two it disappeared entirely. Several of the patients in whom atropia failed were near death when it was given. In the eight cases of rheumatism the atrophia gave permanent relief in five; in two it produced a marked diminution of the sweats; in one it was useless. In one of the cases of ulcerative endocarditis it proved useful, not so in the other. In the two cases of trichinosis the cessation of the acute stage of the disease and of the hectic fever attending it, was followed without any rise of temperature by profuse night sweats. Sulphate of atropia in doses of a milligramme (0.15 grain) was given two hours before the expected access of sweating daily, for five days in succession in one case, and for three days in the other; the result being that the sweats entirely disappeared from the first evening it was given. In one of the cases of rheumatism in a man aged 32, nearly all the large joints of the upper and lower limbs had been severely affected.

* British Medical Journal, November 29th, 1873.

during five days; the patient was covered with sudamina, and when seen by Dr. Fräutzel was bathed in sweat. A milligramme of sulphate of atropia was given immediately, and very soon there was an abatement of the sweating which in two hours disappeared. It returned during the night, but ceased the next forenoon after the administration of a similar dose. The atropia was thenceforth given regularly night and morning with the effect of completely preventing the sweating, the fever lasted 14 days. In another case of acute articular rheumatism the atropia was given first in doses of one, then of two milligrammes with a similar result, and it is remarked that, on two days in the course of the disease on which it was omitted, the sweating returned. The atropia was given according to the following formula: ℞. Sulphate atropia, $\frac{9}{100}$ of a grain; extract of gentian, sufficient to make 10 pills. Dr. Fräutzel has never given larger doses than $\frac{1}{2}$ milligramme (a little less than $\frac{1}{50}$ of a grain) from fear of producing toxic symptoms. In not a few cases, after taking the medicine the patient felt itching in the neck, which however, disappeared in one or two hours. The pupils acted slowly and were sometimes dilated, and in some cases there was muscæ-volitantes. The atropia had to be stopped in four cases on account of diarrhœa, that this was due to the medicine was proved by the fact that it ceased when the atropia was discontinued and reappeared when it was resumed. What the physiological action of atropia is, in arresting sweating, it is difficult to determine. These profuse sweats arise from a relaxation of the walls of the vessels supplied to the sudoriparous glands, and we know that atropia contracts the smallest of these, hence its administration is followed by a reduction of the perspiration, which must continue so long as the system is under the influence of the atropia. If the remedy be continued until the walls of the vessels regain their tonicity it follows naturally that the disease must be conquered. But, some may say, of what use is a remedy that will not entirely remove the disease? We might with the same propriety ask why it is that we continue the use of quinine day after day in the treatment of intermittent fevers, when one large dose will break the force of the paroxysm. The fact that the sweating returns after the effects of the atropia has passed off, and not until then, is an indication to us to push the remedy until we have conquered the disease.

I will add one report of two cases which have recently come under my own observation. Mr. G. who is suffering from phthisis pulmonalis, begged me to give him something to stop his sweating so profusely every night. I prescribed for him the following: ℞. Atropia sulphas, gr. ss; extractum gentiana alc., ʒi. M. ft. pil No. xxx., of which one was to be taken each night. The first night after commencing their use, his sweating was much diminished and the next it did not appear at all, nor has he been troubled since, although he has taken but sixteen of the pills, and another benefit which he derived from their use was, that his bowels which had been obstinately constipated, were so far improved that he now has one or two passages each day.

Case 2.—Mr. B., who from childhood has been troubled with profuse sweating in the arm pits, so that, (unless he wore a pad,) he would spoil a cloth coat in a very short time, staining it so that it was not fit to be worn. I prescribed for him: ℞. Atropia sulphas, grs. ij; ad. ℥i. M. To be applied to the arm pits, twice daily, by sponging. Under the use of the remedy the sweating was stopped, or at least so changed in character and smell that it ceased to annoy him. Each of us have chances almost daily to try this agent, and I hope to see reports from time to time from those under whose observations this may fall, for by these means is knowledge promulgated, and our profession advanced.

